# Native T1 is independently associated with aerobic exercise capacity in long-term follow-up after mild initial COVID-19 disease (Impression COVID&Heart Study)

**DOI:** 10.1016/j.jocmr.2026.102725

**Published:** 2026-04-15

**Authors:** Monika Rozewicz-Juraszek, Stephan Mueller, Midjisuren Ganbat, Carlos Rodriguez Bolanos, Anna Klement, Sonia Antoñana Ugalde, Martin Halle, Eike Nagel, Valentina O. Puntmann

**Affiliations:** a Institute for Experimental and Translational Cardiovascular Imaging, DZHK Centre for Cardiovascular Imaging, Goethe University, Frankfurt am Main, Germany; b Technical University of Munich, TUM School of Medicine and Health, Department for Preventive Sports Medicine and Sports Cardiology, Munich, Germany; c DZHK (German Centre for Cardiovascular Research), Partner Site Munich Heart Alliance, Munich, Germany; d DZHK (German Centre for Cardiovascular Research) Centre for Cardiovascular Imaging, partner site Rhein-Main, Frankfurt am Main, Germany

**Keywords:** Long COVID, Cardiopulmonary exercise testing, VO₂peak, Cardiovascular magnetic resonance, Native T1 mapping, Effort intolerance

## Abstract

**Background:**

Exercise intolerance is a common and incapacitating long-term consequence of coronavirus disease (COVID-19), even after mild acute illness. Cardiovascular magnetic resonance (CMR) studies have demonstrated persistent perimyocardial inflammatory abnormalities; however, their relationship with long-term aerobic capacity remains unclear.

**Methods:**

In this prospective observational study, individuals without prior structural heart disease underwent standardized CMR, echocardiography, and cardiopulmonary exercise testing (CPET) at least 3 years after the initial COVID-19. The primary endpoint was the association between %-predicted peak oxygen consumption (VO₂peak) (age-sex-body mass index (BMI) adjusted, Study of Health in Pomerania (SHIP) https://doi.org/10.1007/s00103-012-1483-6) and imaging parameters. Secondary analyses included lactate measurements and sex-stratified models.

**Results:**

A total of 132 participants (mean age 49±12 years; 68/132 (52%) male) were evaluated 48 months [interquartile range (IQR) 42–53] post-infection. Non-ischemic perimyocardial enhancement was present in 34/132 (26%), whereas two participants had an unrecognized ischemic scar. Male sex, higher BMI, lower age, and higher native T1 were associated with lower %-predicted VO₂peak in univariate models. In multivariate analysis, male sex, lower age, and higher native T1 (β = −0.25 per ms, 95% CI −0.40 to −0.10; p<0.001) remained independent predictors of lower %-predicted VO₂peak. In total, 63/132 (48%) participants demonstrated %-predicted VO₂peak below predicted values. Sex-stratified multivariate analyses showed that higher native T1 (p<0.001) independently associated with lower %-predicted VO₂peak in both men and women, with lower age additionally retained in men. In a lactate-measured subgroup (n = 73), higher resting lactate, higher native T1, male sex, and lower left atrial area were associated with lower %-predicted VO₂peak.

**Conclusion:**

In long-term follow-up of individuals with mild initial COVID-19 and no prior structural heart disease, aerobic capacity relative to predicted values was reduced in 48% of participants, particularly in men, and was independently associated with higher myocardial native T1.

## Graphical abstract

**Figure:**
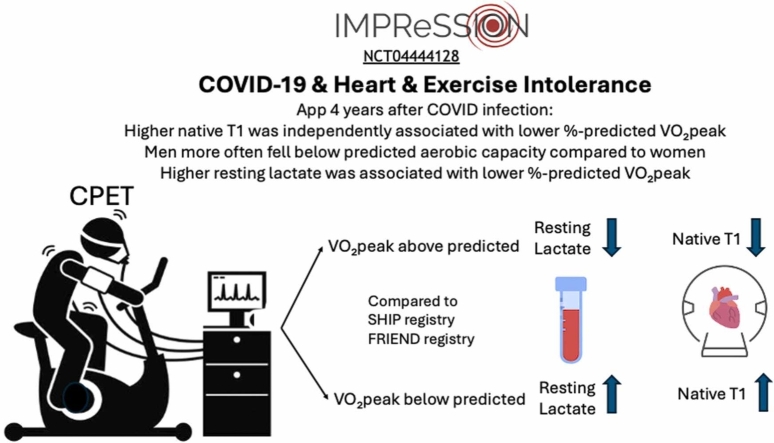

## 1 Introduction

Exercise intolerance has emerged as a common long-term sequela of COVID-19, even among individuals with mild acute disease 1, 2, 3, 4. Proposed mechanisms include persistent inflammatory microvascular and endothelial dysfunction, resulting in peripheral hypoperfusion, altered mitochondrial function, and abnormal metabolic responses to exercise 5, 6. Studies using cardiopulmonary exercise testing (CPET) have consistently demonstrated reduced peak oxygen consumption (VO₂peak), indicating reduced aerobic capacity in post-acute sequelae of COVID-19 (PASC), with meta-analyses confirming lower values compared with control populations [7]. Metabolic studies have further suggested impaired substrate utilization and altered lactate dynamics during exercise, implicating disrupted peripheral vascular oxygen delivery and reduced anaerobic threshold in the pathogenesis of reduced exercise capacity [5]. Sex, cardiovascular risk factors, and pre-COVID fitness have all been shown to modify the severity of the functional limitation 8, 9, 10. Paradoxically, physical activity and training can worsen symptoms, a phenomenon known as post-exercise malaise (PEM) [11]. As individuals adopt activity avoidance in response to post-exertional symptom exacerbation, this may secondarily contribute to cardiovascular deconditioning in the long term.

Cardiovascular imaging has provided insights into the inflammatory involvement of the cardiovascular system in PASC. Most patients show subtle abnormalities of systolic and diastolic function on transthoracic echocardiography (TTE), and signs of pericardial inflammation, even in those with persistent symptoms [12,^13^, ^14^, ^15^. Cardiovascular magnetic resonance (CMR) studies consistently revealed non-ischemic inflammatory perimyocardial patterns of late gadolinium enhancement (LGE) [16]. Significant structural heart disease due to a profoundly necrotic myocarditis is rare in individuals with mild initial disease. Native T1, female sex, and E/e′ were associated with persistent symptoms in short-term follow-up approximately a year post-infection 13, 17. However, the relationship between the above cardiac imaging findings and long-term exercise capacity in individuals with mild initial COVID illness remains unknown. We hypothesized that diffuse subclinical myocardial inflammation is associated with reduced aerobic exercise capacity (VO₂peak) at least 3 years after the initial infection in individuals with mild initial illness and no prior structural heart disease.

## 2 Methods

The study population and the design of the Impression COVID&Heart Study have been described previously 13, 17, 18. In brief, this is a prospective, single-center, observational cohort study of previously well individuals at baseline, without known cardiovascular disease, other significant comorbidities, long-term guideline-directed cardiovascular pharmacotherapy (including antihypertensive agents and other cardiovascular medications), or a formal clinical indication for CMR imaging. Eligibility was confirmed by normal blood test results at study inclusion, including troponin, and by the absence of structural heart disease on CMR with contrast agent (ClinicalTrials.gov: NCT04444128). The overarching research objective is to characterize early pathophysiological transitions from health to subclinical cardiovascular disease through comprehensive cardiac imaging and tissue characterization.

The present analysis reports on the outcomes of the Impression COVID&Heart Substudy of consecutive adult participants (18 years of age or older) with documented mild initial COVID-19 infection, who underwent baseline eligibility assessments between April 2020 and October 2021. Follow-up assessments were performed at least 3 years after the index infection and included cardiac imaging with CMR and TTE, and CPET, all conducted on the same day. The study protocol was approved by the Ethics Committee of the Goethe University Hospital Frankfurt, Germany (No. 463/16). Written informed consent was obtained from all participants.

## 3 Image acquisition and postprocessing

### 3.1 Cardiovascular magnetic resonance

All CMR examinations were performed on a clinical 3T scanner (Magnetom Skyra, Siemens Healthineers, Erlangen, Germany) using a standardized imaging protocol comprising cine imaging for cardiac volumes, mass and function, native T1 and T2 mapping, myocardial perfusion using vasodilatory agent (regadenoson, Rapiscan 400 mcg bolus, GE Healthcare, Chicago, Illinois), and LGE imaging (gadobutrol 0.1 mmol/kg, Gadovist, Bayer Healthcare, Leverkusen, Germany). The details of all imaging parameters were reported previously 13, 17, 18.

All imaging staff underwent standardized training in imaging procedures with regular quality control assessments. Slice thickness was set uniformly at 8 mm. Image quality was assessed during acquisition, with particular attention to artefacts affecting mapping sequences. Significant myocardial ischemia, defined as ≥10% of ischemic myocardium within the territory of a coronary artery [19], was excluded by visual assessment of myocardial perfusion imaging. Postprocessing was performed by the study core laboratory, blinded to the clinical information. Ventricular volumes, mass, and function were quantified using automated contour detection software with manual correction (SuiteHeart, Neosoft LLC, Pewaukee, Wisconsin), according to standard postprocessing recommendations [20]. All volume measurements are normalized to body surface area. Native T1 and T2 mapping values were measured in the septal myocardium of the midventricular short-axis slice using motion-corrected images. Areas of LGE were excluded from the relaxation measurements to avoid contamination by replacement scar. Hematocrit was not routinely measured, and ECV was not calculated. Interpretation of LGE images followed standardized postprocessing recommendations [20]. Myocardial LGE was visually defined by two observers based on the presence and predominant pattern as ischemic or non-ischemic. Non-ischemic perimyocardial enhancement pattern was determined visually and reported as present or absent in a binary fashion.

### 3.2 Transthoracic echocardiography

TTE was performed using a commercially available system (Vivid E95, GE Healthcare) in accordance with the recommendations of the World Alliance Societies of Echocardiography 21, 22. Images were analyzed offline with EchoPAC (version 203, GE Vingmed Ultrasound, Chicago, Illinois). Based on prior findings [13], diastolic function was characterized using E/e′, calculated as the average of septal and lateral measurements, derived from transmitral Doppler inflow and tissue Doppler velocities.

### 3.3 Cardiopulmonary exercise testing

CPET was performed on an electronically braked cycle ergometer (Cardiovit CS-200 Office Spiroergometry, Schiller, Feldkirchen, Germany) following current guidelines [23]. Equipment calibration and testing procedures adhered to manufacturer specifications. Participants wore a tight-fitting nonrebreathing mask connected to a metabolic cart with gas sensors, with continuous electrocardiographic (ECG) and blood pressure (BP) monitoring. After a resting period with baseline 12-lead ECG and BP, exercise was performed using an individualized ramp protocol consisting of a 3-minute warm-up at 20–30 W, followed by incremental workload increases targeting volitional exhaustion within 8–12 min, and subsequent recovery. Cadence was maintained above 55 rpm, and participants were encouraged to achieve maximal effort. Breath-by-breath gas exchange, ventilatory parameters, and operating lung volumes (via inspiratory capacity maneuvers) were recorded, with flow–volume loops obtained at rest, at the end of warm-up, and at peak exercise. The datasets were analyzed in a blinded manner at the study core laboratory in Munich [24]. VO_2_ peak was defined as the highest 30-second VO_2_ average during the test. Submaximal effort was defined by attainment of <80% of the age-sex predicted maximal HR (220 bpm – age), or a peak respiratory exchange ratio (RER) of <1.15.

Chest CT was not part of the study protocol. All participants underwent standardized lung function assessment, including spirometry and flow–volume loop analysis obtained at rest, at the end of the warm-up phase, and at peak exercise, as an integral component of the CPET protocol. The presence of significant pulmonary disease constitutes a contraindication for CPET participation and was an exclusion criterion at enrollment; none of the participants demonstrated evidence of significant pulmonary impairment.

### 3.4 Statistical analysis

The primary outcome was %-predicted VO₂peak (calculated as measured VO₂peak relative to age-, sex-, and BMI-adjusted predicted values from the SHIP and Fitness Registry and the Importance of Exercise National Database [FRIEND] datasets) 25, 26. Associations between %-predicted VO₂peak and clinical, imaging, and metabolic variables were first explored using univariable linear regression analyses (Supplementary material). Multivariable linear regression models were then constructed using variables defined a priori based on physiological relevance and study design, including age, sex, body mass index (BMI), left ventricular end-diastolic volume index (LV-EDVi), left atrial area, native T1, and native T2. Analyses were additionally stratified by sex. A secondary analysis was performed in the subgroup with available lactate measurements at rest and peak exercise. To align with the SHIP reference population, sensitivity analyses were conducted after excluding participants with BMI ≥30 kg/m² or current smoking. Further sensitivity analyses excluded participants with cardiovascular risk factors at the follow-up visit (hypertension, n = 14, prediabetes, n = 3). To assess robustness across reference standards, additional analyses were performed using predicted VO₂peak values derived from the FRIEND registry 25, 26.

Normality of distribution for continuous variables was assessed using the Shapiro–Wilk test. Continuous variables are presented as mean ± standard deviation (SD) or median [interquartile range, IQR], and categorical variables as counts (%). Group comparisons used the Wilcoxon rank-sum test for continuous and Pearson χ² or Fisher’s exact test for categorical variables. Model estimates are reported as regression coefficients (β) with 95% confidence intervals. Correction for multiple testing was performed using false discovery rate adjustment where applicable. Statistical analysis was performed using R (RStudio Version 2025.09.0; RStudio Inc. with R packages dplyr, gtsummary, ggplot2, and stats).

## 4 Results

**Fig. 1:**
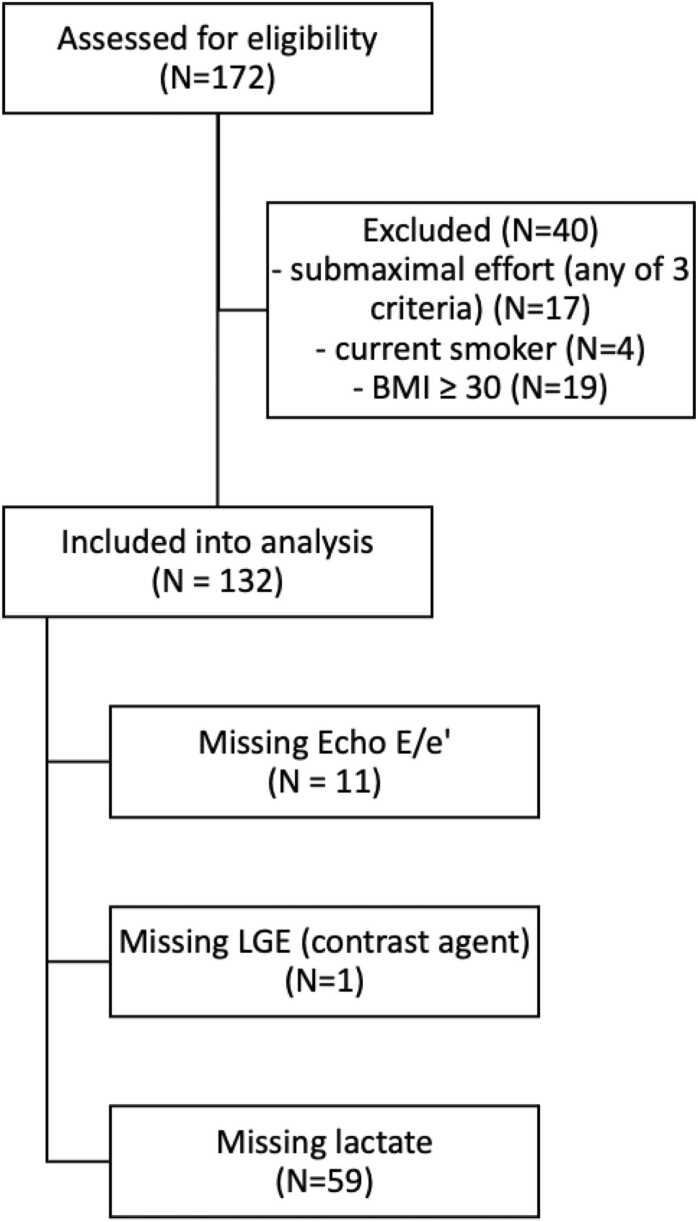
CONSORT flow diagram. *BMI* body mass index, *CPET* cardiopulmonary exercise testing, *CMR* cardiovascular magnetic resonance, *RER* respiratory exchange ratio, *VO₂peak* peak oxygen uptake

Baseline characteristics for the full cohort are provided in Table 1, with sex-specific characteristics presented in Table 2A, Table 2B. Of 172 consecutive participants assessed, 40 were excluded (17 due to submaximal effort as defined above, 4 due to current smoking and 19 due to a BMI of ≥30 kg/m^2^), resulting in a final analytical cohort of 132 participants (mean age 49±12 years; 68/132 (52%) male (Fig. 1). The median time since the index COVID-19 infection was 47.9 months [IQR 41.9, 53.3]. For descriptive purposes, participants were stratified according to %-predicted VO₂peak derived from the SHIP reference dataset into those below predicted values (<100%; n = 63) and those at or above predicted values (≥100%; n = 69). Male sex was over-represented among participants with %-predicted VO₂peak <100 (n = 64, 65%) and under-represented among those ≥100 (33%). Participants achieving values ≥100 were slightly older (p = 0.025), an effect driven predominantly by men (Table 2A).

**Table 1:** Demographics, imaging, and CPET values of the overall cohort, and stratified for predicted VO₂peak from the SHIP dataset [1]

Characteristic	Overall N = 132^a^	Below predicted N = 63^a^	Above predicted N = 69^a^	p-value^b^
Age (y)	49.3±11.9	46.9±12.5	51.4±11.0	0.025
*Gender*				<0.001
Male n, %	64 (48%)	41 (65%)	23 (33%)	
Female n, %	68 (52%)	22 (35%)	46 (67%)	
BMI kg/m^2^	24.5±2.80	25.2±2.80	23.8±2.70	0.007
months since first COVID infection	47.9 [41.9, 53.3]	48.3 [43.5, 52.3]	47.0 [39.0, 53.9]	0.61
Hypertension n, %	14 (11%)	8 (13%)	6 (8.7%)	0.46
Diabetes n, %	3 (2.3%)	2 (3.2%)	1 (1.4%)	0.51
BPsys (mmHg)	125.3±16.5	127.3±16.3	123.3±16.6	0.20
BPdia (mmHg)	80.9±10.3	81.3±9.90	80.5±10.8	0.48
HR (bpm)	67.6±11.0	68.0±11.7	67.3±10.3	0.60
LV-EDVi (mL/m²)	85.0±13.0	85.2±14.0	84.7±12.2	0.46
LV-ESVi (mL/m²)	36.7±7.80	36.7±8.00	36.6±7.70	0.44
Svi (mL/m²)	48.3±7.60	48.7±8.20	48.0±7.00	0.62
LVEF (%)	56.7±6.40	57.2±4.80	56.3±7.60	0.79
RVEF (%)	56.3±6.00	56.4±6.10	56.3±5.90	0.91
LA Area (cm²)	24.2±4.10	23.9±3.80	24.5±4.50	0.39
E/e′	6.40±1.70	6.20±1.60	6.60±1.80	0.13
*LGE presence and type*				0.084
None n, %	92 (71%)	39 (64%)	53 (78%)	
Ischemic n, %	2 (1.6%)	0 (0%)	2 (2.9%)	
Non-ischemic n, %	34 (26%)	21 (34%)	13 (19%)	
native T1 (ms)	1128.0±26.9	1134.6±28.6	1122.0±23.9	0.008
native T2 (ms)	38.1±1.40	38.3±1.40	38.0±1.40	0.51
VO₂max (mL/kg/min)	28.4±5.70	25.8±5.50	30.7±4.80	<0.001
venous lactate rest (mg/dl)	11.5±5.00	12.5±5.80	10.6±4.20	0.13
venous lactate exercise (mg/dl)	41.7±21.8	48.2±24.9	35.7±16.9	0.042
%-predicted VO₂peak	99.5±18.4	85.1±14.3	112.6±9.90	<0.001

Lactate values relate to a subgroup of patients (n = 73), *BMI* body mass index, *BP* blood pressure, *HR* heart rate, *LV* left ventricle, *EDVi* index end-diastolic volume, *ESVi* indexed end-systolic volume, *Svi* stroke volume index, *RV* right ventricle, *EF* ejection fraction, *LA* left atrium, *VO₂peak* peak oxygen uptake.

a Mean ± SD; n (%); Median [Q1, Q3]

b Wilcoxon rank sum test; Pearson’s Chi-squared test; NA

**Table 2A:** Demographics, imaging, and CPET values of the overall male cohort, and stratified for predicted VO₂peak from the SHIP dataset [25]

Characteristic	Overall N = 64^a^	Below predicted N = 41^a^	Above predicted N = 23^a^	p-value^b^
Age (y)	48.5±12.7	45.9±12.7	53.1±11.4	**0.012**
BMI (kg/m^2^)	25.5±2.40	25.7±2.50	25.2±2.30	0.26
Duration since first COVID-19 infection (months)	47.5 [42.9, 54.2]	46.8 [43.5, 50.6]	51.5 [39.0, 62.8]	0.21
Hypertension n, %	6 (9.4%)	4 (9.8%)	2 (8.7%)	0.89
Diabetes n, %	3 (4.7%)	2 (4.9%)	1 (4.3%)	0.92
BPsys (mmHg)	30.9±14.4	131.1±14.6	130.5±14.4	0.79
BPdia (mmHg)	82.7±9.20	82.4±9.30	83.3±9.20	0.85
HR (bpm)	67.5±11.9	67.8±12.2	66.9±11.5	0.69
LVEF (%)	56.1±4.50	55.9±4.60	56.5±4.50	0.47
LV-EDVi (mL/m²)	90.5±11.9	90.5±11.8	90.6±12.5	0.63
LV-ESVi (mL/m²)	40.1±7.20	40.0±6.50	40.3±8.50	0.60
SVi (mL/m²)	50.6±7.60	50.7±7.80	50.4±7.40	0.83
RVEF (%)	53.6±5.20	54.3±5.70	52.2±3.90	0.15
LA Area (cm²)	25.7±4.10	25.0±3.50	26.8±4.80	0.15
E/e′	6.20±1.7 (N=61)	6.20±1.7 (N=38)	6.2±1.8	0.64
*LGE presence and type*				0.063
none n, %	42 (67%)	26 (63%)	16 (73%)	
ischemic n, %	2 (3.2%)	0 (0%)	2 (9.1%)	
non-ischemic n, %	19 (30%)	15 (37%)	4 (18%)	
native T1 (ms)	1125.2±28.5	1128.6±28.1	1119.1±28.7	0.17
native T2 (ms)	38.0±1.30	38.1±1.20	38.0±1.40	0.87
VO₂peak (mL/kg/min)	30.1±6.10	27.5±5.60	34.6±4.00	<0.001
%-predicted VO₂peak (mL/kg/min)	92.7±18.4	83.2±16.1	109.6±5.90	<0.001
venous lactate rest (mg/dl)	12.7±5.8 (N=36)	13.6±6.5 (N=23)	11.2±3.8 (N=13)	0.39
venous lactate exercise (mg/dl)	46.2±24.8 (N=36)	50.3±28.1 (N=23)	39.0±16.0 (N=13)	0.38

Lactate values relate to a subgroup of patients (n = 36), *BMI* body mass index, *BP* blood pressure, *HR* heart rate, *LV* left ventricle, *EDVi* index end-diastolic volume, *ESVi* indexed end-systolic volume, *Svi* stroke volume index, *RV* right ventricle, *EF* ejection fraction, *LA* left atrium, *VO₂peak* peak oxygen uptake.

a Mean ± SD; n (%); Median [Q1, Q3]

b Wilcoxon rank sum test; Pearson’s Chi-squared test

**Table 2B:** Demographics, imaging, and CPET values of the overall female cohort, and stratified for predicted VO₂peak from the SHIP dataset [25]

Characteristic	Overall N = 68^a^	Below predicted N = 22^a^	Above predicted N = 46^a^	p-value^b^
Age (y)	50.0±11.3	48.7±12.2	50.6±10.9	0.64
BMI (kg/m^2^)	23.6±2.90	24.3±3.10	23.2±2.70	0.24
Duration since first COVID-19 infection (months)	47.9 [41.0, 53.0]	50.5 [43.8, 54.6]	45.9 [38.8, 52.4]	0.090
Hypertension n, %	8 (12%)	4 (18%)	4 (8.7%)	0.26
Diabetes n, %	0 (0%)	0 (0%)	0 (0%)	
BPsys (mmHg)	119.7±16.7	120.0±17.3	119.5±16.6	0.93
BPdia (mmHg)	79.0±11.1	79.1±11.0	78.9±11.3	>0.99
HR (bpm)	67.8±10.1	68.5±11.0	67.5±9.70	0.63
LVEF (%)	57.3±7.80	59.6±4.50	56.2±8.80	0.041
LV-EDVi (mL/m²)	79.7±11.9	75.4±12.7	81.8±11.1	0.057
LV-ESVi (mL/m²)	33.5±7.00	30.6±7.00	34.9±6.70	0.022
SVi (mL/m²)	46.2±7.00	44.9±7.80	46.8±6.50	0.47
RVEF (%)	59.0±5.50	60.3±5.00	58.3±5.60	0.19
LA Area (cm²)	22.8±3.70	21.6±3.10	23.4±3.90	0.048
E/e′	6.60±1.7 (N=60)	6.20±1.3 (N=18)	6.80±1.8 (N=42)	0.18
*LGE presence and type*				0.16
None n, %	50 (76%)	13 (65%)	37 (80%)	
Ischemic n, %	0 (0%)	0 (0%)	0 (0%)	
non-ischemic n, %	15 (23%)	6 (30%)	9 (20%)	
native T1 (ms)	1130.7±25.3	1145.9±26.6	1123.4±21.3	0.003
native T2 (ms)	38.2±1.50	38.6±1.70	38.0±1.40	0.21
VO₂peak (mL/kg/min)	26.8±4.80	22.5±3.50	28.8±3.90	<0.001
%-predicted VO₂peak (mL/kg/min)	105.9±16.0	88.6±9.60	114.1±11.2	<0.001
venous lactate rest (mg/dl)	10.3±3.9 (N=37)	10.1±2.4 (N=11)	10.4±4.4 (N=26)	0.65
venous lactate exercise (mg/dl)	37.1±17.6 (N=37)	43.8±16.7 (N=11)	34.0±17.4 (N=26)	0.13

Lactate values relate to a subgroup of patients (n = 36), *BMI* body mass index, *BP* blood pressure, *HR* heart rate, *LV* left ventricle, *EDVi* index end-diastolic volume, *ESVi* indexed end-systolic volume, *Svi* stroke volume index, *RV* right ventricle, *EF* ejection fraction, *LA* left atrium, *VO₂peak* peak oxygen uptake.

a Mean ± SD; n (%); Median [Q1, Q3]

b Wilcoxon rank sum test; Pearson’s Chi-squared test

The groups were similar for cardiac structure and function (Table 1). In sex-specific analyses, women with %-predicted VO₂peak <100 demonstrated smaller LV end-systolic volume index (ESVi) and LA area, and higher LVEF compared with women ≥100% predicted (Table 2B). Native T1 was significantly higher in women with %-predicted VO₂peak <100 (p = 0.003). Non-ischemic perimyocardial enhancement was present in 34/132 (26%) participants, while 2/132 (2%) exhibited previously unrecognized ischemic LGE. LGE prevalence and pattern did not differ between %-predicted VO₂peak groups.

**Fig. 2:**
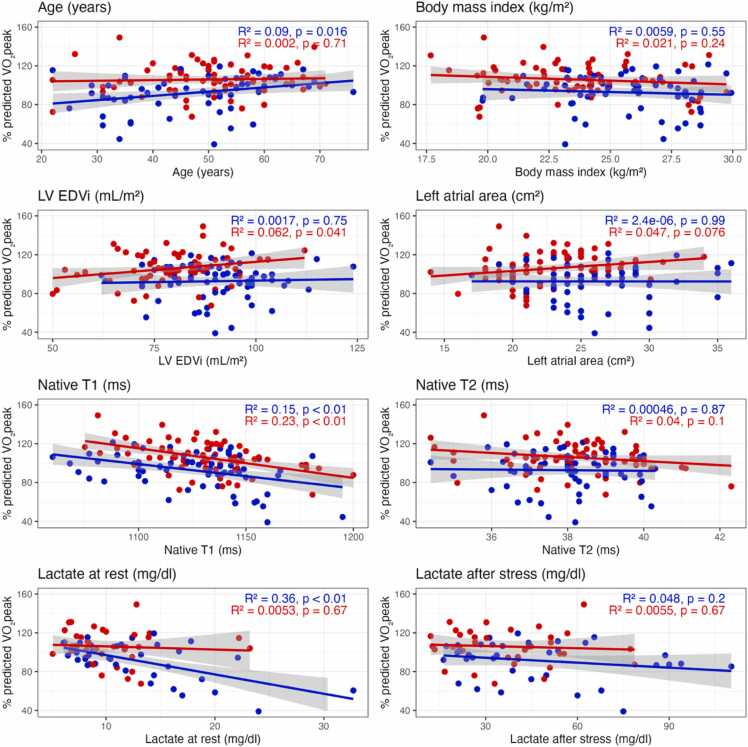
Scatter plots of prespecified covariates versus %-predicted VO₂peak with sex-coded points (male = blue; female = red). Solid lines indicate overall OLS regression fits with 95% confidence intervals; in-panel values denote R² and slope p-values. Resting lactate plots are shown for the lactate subgroup only (n = 73). The distribution illustrates a lower %-predicted VO₂peak in men and inverse associations with myocardial native T1 and resting lactate. *BMI* body mass index, *OLS* ordinary least squares, *T1* native myocardial T1 relaxation time, *VO₂peak* peak oxygen uptake, *LV* left ventricle, *EDV* end-diastolic volume

On average, men demonstrated measured VO₂peak values below predicted norms, whereas women exceeded predicted values (Fig. 2). Venous lactate measurements at rest and stress were available in a subgroup of 73 participants. In unadjusted comparisons, peak exercise lactate was higher in participants with %-predicted VO₂peak <100 (p = 0.042).

### 4.1 Associations with %-predicted VO₂peak

In univariable linear regression analyses of the full cohort (Table 3), male sex, lower age, higher BMI, and higher native T1 were associated with lower %-predicted VO₂peak. In the multivariable model, male sex (p<0.001), lower age (p = 0.016) and higher native T1 (β = −0.25 per ms, 95% CI −0.40 to −0.10; p<0.001) remained independently associated with lower %-predicted VO₂peak (model R^2^ 0.35; adjusted R^2^ 0.32).

**Table 3:** Univariable and multivariable analyses of %-predicted VO₂peak from the SHIP dataset [25] at 47 months of follow-up for the whole cohort (n = 132; for the model: R^2^ 0.35; adj. R^2^ 0.315)

	Univariable				Multivariable		
Characteristic	Β	95% CI	p-value	q-value^a^	Β	95% CI	p-value
Age (y)	0.30	0.04, 0.56	**0.025**	0.092	0.29	0.06, 0.53	**0.038**
BMI (kg/m²)	−1.4	−2.5, −0.33	**0.011**	0.061	0.27	−0.83, 1.4	0.6
Gender (male)	13	7.2, 19	**<0.001**	0.001	18	12, 25	<0.001
LVEF (%)	0.04	−0.45, 0.54	0.9	0.9			
LV EDVi (mL/m²)	−0.04	−0.29, 0.20	0.7	0.8	0.18	−0.08, 0.44	0.2
LV SVi (mL/m²)	−0.07	−0.49, 0.35	0.7	0.8			
LVMi (g/m²)	−0.23	−0.53, 0.07	0.14	0.4			
RVEF (%)	0.09	−0.45, 0.62	0.7	0.8			
LA area (cm²)	−0.17	−0.95, 0.60	0.7	0.8	0.60	−0.15, 1.4	0.
E/e′	1.4	−0.61, 3.4	0.2	0.4			
Native T1 (ms)	−0.25	−0.36, −0.14	**<0.001**	**<0.001**	−0.29	−0.40, −0.17	**<0.001**
Native T2 (ms)	−1.1	−3.3, 1.2	0.3	0.6	0.45	−2.4, 1.6	0.7

*CI* confidence interval, *BMI* body mass index, *BP* blood pressure, *HR* heart rate, *LV* left ventricle, *EDVi* index end-diastolic volume, *ESVi* indexed end-systolic volume, *Svi* stroke volume index, *RV* right ventricle, *EF* ejection fraction, *LA* left atrium, *VO₂peak* peak oxygen uptake.

a False discovery rate correction for multiple testing

In the subgroup with available lactate measurements (n = 73), higher resting lactate was strongly associated with lower %-predicted VO₂peak (β = −1.4 per mg/dl, −2.3 to −0.62; p<0.001). In the multivariable model, male sex (p = 0.015), smaller LA area (p = 0.041), higher native T1 (p = 0.008), and higher resting lactate (p<0.01) remained independently associated with reduced %-predicted VO₂peak (model R² = 0.482; adjusted R² = 0.405). Peak exercise lactate was not retained in adjusted models. Scatterplots illustrating associations between %-predicted VO₂peak and prespecified covariates are shown in Fig. 2.

### 4.2 Sex-stratified analyses

Among men (n = 64), lower age (p = 0.016) and higher native T1 (p<0.002) were associated with lower %-predicted VO₂peak in univariable analyses and remained independent predictors in the multivariable model (model R² = 0.261; adjusted R² = 0.182; Table 4). In the lactate-measurements male subgroup, higher resting lactate (p<0.001) was the sole independent predictor of reduced %-predicted VO₂peak in the multivariable model(R^2^ 0.557, adjusted R^2^ 0.421).

**Table 4:** Univariable and multivariable analyses of %-predicted VO₂peak from the SHIP dataset [25] at 47 months of follow-up for males (n = 64; for the model: R^2^ 0.261; adj. R^2^ 0.182)

	Univariable				Multivariable		
Characteristic	Β	95% CI	p-value	q-value^a^	Β	95% CI	p-value
Age (y)	0.44	0.09, 0.79	**0.016**	0.087	0.53	0.15, 0.91	**0.008**
BMI (kg/m²)	−0.58	−2.5, 1.3	0.5	0.8	0.06	−1.8, 2.0	<0.9
LVEF (%)	0.50	−0.54, 1.5	0.3	0.8			
LV EDVi (mL/m²)	0.06	−0.33, 0.46	0.7	0.8	0.24	−0.16, 0.65	0.2
LV SVi (mL/m²)	0.11	−0.51, 0.72	0.7	0.8			
LVMi (g/m²)	0.24	−0.25, 0.73	0.3	0.8			
RVEF (%)	−0.40	−1.3, 0.49	0.4	0.8			
LA area (cm²)−	0.01	−1.2, 1.2	>0.9	>0.9	0.15	−1.0, 1.3	0.8
E/e′	0.72	−2.1, 3.6	0.6	0.8			
Native T1 (ms)	−0.25	−0.40, −0.10	**0.002**	**0.018**	-0.27	−0.44, −0.10	**<0.003**
Native T2 (ms)	−0.31	−4.0, 3.4	0.9	>0.9	0.58	−2.9, 4.1	>0.7

*CI* confidence interval, *BMI* body mass index, *BP* blood pressure, *HR* heart rate, *LV* left ventricle, *EDVi* index end-diastolic volume, *ESVi* indexed end-systolic volume, *Svi* stroke volume index, *RV* right ventricle, *EF* ejection fraction, *LA* left atrium, *VO₂peak* peak oxygen uptake.

a False discovery rate correction for multiple testing

Among women (n = 68), higher native T1 (p<0.001) and lower LV-EDVi (p = 0.041) were associated with lower %-predicted VO₂peak in the univariable analysis. In multivariable analyses, native T1 remained the only independent predictor (p<0.003; model R² = 0.261, adjusted R² = 0.182, Table 5). In the lactate measurements female subgroup, native T1 again emerged as the only independent predictor (p = 0.025; model R² = 0.402; adjusted R² = 0.218).

**Table 5:** Univariable and multivariable analyses of %-predicted VO₂peak from the SHIP dataset [25] at 47 months of follow-up for females (N = 68; for the model: R^2^ 0.322; adj. R^2^ 0.255)

	Univariable				Multivariable		
Characteristic	Β	95% CI	p-value	q-value^a^	Β	95% CI	p-value
Age (y)	0.06	-0.28, 0.41	0.7	0.7	0.10	-0.22, 0.41	0.5
BMI (kg/m²)	-0.79	-2.1, 0.55	0.2	0.4	0.39	-0.96, 1.7	0.6
LVEF (%)	-0.22	-0.72, 0.28	0.4	0.4			
LV EDVi (mL/m²)	0.34	0.01, 0.66	**0.041**	0.2	0.19	-0.16, 0.54	0.3
LV SVi (mL/m²)	0.30	-0.26, 0.86	0.3	0.4			
LVMi (g/m²)	0.23	-0.30, 0.76	0.4	0.4			
RVEF (%)	-0.62	-1.3, 0.08	0.081	0.2			
LA area (cm²)	0.93	-0.10, 2.0	0.076	0.2	0.98	-0.02, 2.0	0.054
E/e′	1.1	-1.4, 3.6	0.4	0.4			
Native T1 (ms)	-0.30	-0.44, −0.17	**<0.001**	<0.001	-0.29	-0.45, −0.14	**<0.001**
Native T2 (ms)	-2.1	-4.6, 0.43	0.1	0.2	-1.2	-3.6, 1.3	0.3

*CI* confidence interval, *BMI* body mass index, *BP* blood pressure, *HR* heart rate, *LV* left ventricle, *EDVi* index end-diastolic volume, *ESVi* indexed end-systolic volume, *Svi* stroke volume index, *RV* right ventricle, *EF* ejection fraction, *LA* left atrium, *VO₂peak* peak oxygen uptake.

a False discovery rate correction for multiple testing

### 4.3 Reference and sensitivity analyses

Parallel analyses using %-predicted VO₂peak derived from the FRIEND reference dataset demonstrate consistent direction and magnitude of the associations, with native T1 remaining the principal imaging predictor across all models (Supplementary Tables S1–6).

Sensitivity analyses excluding participants with risk factors at follow-up yielded similar results, with native T1 remaining independently associated in all analyses except in the male lactate-measured subgroup (data not shown).

## 5 Discussion

In individuals without prior heart conditions, assessed nearly four years after their mild index COVID-19 infection, aerobic exercise capacity relative to predicted values was frequently impaired, particularly among men. Lower %-predicted VO₂peak was independently associated with male sex, younger age, and higher native T1, with consistent findings in the sex-stratified analyses. In a subgroup with available metabolic data, higher resting lactate, but not peak exercise lactate, emerged as the strongest metabolic correlate of reduced aerobic capacity.

Native T1 was the principal imaging marker that associated with reduced performance. Higher native T1 values correlated with a lower %-predicted VO₂peak in univariate analyses and remained independently associated in multivariate and sex-stratified models, even when ventricular volumes and ejection fractions were within normal ranges. In contrast, mild variations in conventional structural parameters, such as LV end-diastolic volume index or left atrial area, contributed little explanatory value. These findings suggest that classical measures of cardiac structure and function may be insufficiently sensitive to capture pathophysiologically relevant subclinical myocardial alteration, consistent with prior studies reporting preserved chamber dimensions and diastolic indices despite persistent symptoms and reduced functional capacity 13, 28, 29.

Non-ischemic perimyocardial enhancement was observed in approximately one-quarter of participants, whereas ischemic scar was rare. LGE prevalence did not differ between performance groups and was not retained in multivariable models, suggesting that LGE imaging is a less informative imaging correlate of long-term functional limitation than diffuse myocardial tissue alterations captured by native T1. The inverse association between native T1 and %-predicted VO₂peak, together with normal preload-related indices, argues against plasma volume expansion as a dominant confounder and supports myocardial tissue alterations as the primary correlate. Together, these findings extend prior observations of persistent CMR abnormalities following COVID-19, including elevated tissue mapping values and non-ischemic LGE patterns by demonstrating their relationship to objective functional performance 17, 27.

The effect size for native T1 in the multivariable model (β ≈ −0.25 to −0.30 per ms) indicates that a 10 ms increment in native T1 corresponds to approximately 2.5%–3.0% lower %-predicted VO₂peak. Given that T1 mapping at 3.0T carries a coefficient of variation of approximately 1%–2%, differences of this magnitude approach but do not substantially exceed measurement noise at the individual level and should therefore be interpreted as population-level associations rather than diagnostically discriminating thresholds. The somewhat larger effect observed in women (Table 5) is of potential clinical interest, as T1 elevations at the higher end of the observed range may approach the limit of detectability with serial imaging in dedicated follow-up studies.

A key novel observation of this study is the strong association between higher resting lactate and reduced %-predicted VO₂peak in subgroup analyses. Although exploratory and limited by sample size, this finding is biologically plausible. In healthy cohorts, resting lactate is typically low, and elevations may reflect impaired mitochondrial oxidative metabolism or reduced metabolic efficiency. Previous post-COVID and Myalgic Encephalomyelitis/Chronic Fatigue Syndrome studies have also reported altered lactate dynamics during and after exercise, suggestive of impaired peripheral oxygen utilization, early reliance on anaerobic metabolism, or delayed lactate clearance [2,5].

More recent mechanistic work has demonstrated systemic metabolic disturbances and skeletal muscle abnormalities in long COVID that correlate with reduced exercise capacity, supporting the concept of a persistent metabolic limitation [30]. Our findings extend these observations to long-term follow-up, identifying resting lactate as a marker of metabolic vulnerability associated with reduced aerobic performance.

Across reference frameworks, %-predicted VO₂peak approached population norms on average; however, substantial heterogeneity persisted, with men more frequently falling below predicted values. Native T1 remained the dominant imaging correlate of reduced performance, and higher resting lactate identified a subgroup with disproportionate metabolic limitation. These associations suggest that diffuse myocardial tissue alterations and systemic metabolic signatures may jointly contribute to persistent reductions in exercise capacity years after infection, complementing structural and functional imaging markers.

## 6 Limitations

Several limitations warrant consideration. This observational study is not designed to interrogate causality. Cardiopulmonary exercise testing was incorporated as an amendment to the original protocol, precluding pre-infection or baseline CPET comparisons. Furthermore, no concurrent control group was recruited. This limitation precludes direct case-control comparison and necessitates reliance on external normative reference datasets for VO₂peak benchmarking. The small effect sizes observed for native T1, which are within the measurement variability of standard T1 mapping protocols, should therefore be interpreted with appropriate caution in the absence of a matched control comparator. Accordingly, %-predicted VO₂peak was used as a standardized functional outcome referenced to large, well-established normative cohorts rather than as a case-control discriminator. Residual calibration error in the prediction equation may influence estimates of the specific strata, however, bias was mitigated by applying the identical exclusion criteria to the reference cohorts and replicating analyses using both SHIP and FRIEND registries 25, 26, yielding consistent results. While reference cohorts are necessarily less deeply phenotyped than the present study population, the rigorous eligibility assessment employed here materially reduces the likelihood of unrecognized structural heart disease. Lactate measurements were available only in a subset of participants and should therefore be interpreted as exploratory. The reference cohorts (SHIP and FRIEND) are necessarily less deeply phenotyped than the present study population, and sex-specific calibration differences may influence group-level estimates, particularly for women, in whom predicted values appeared marginally higher than the study-cohort observed values. Finally, although the cohort size is substantial for a single-center study, precision in some subgroup analyses remains limited.

## 7 Conclusions

In individuals without pre-existing heart disease assessed nearly four years after mild initial COVID-19 infection, aerobic exercise capacity relative to predicted values was frequently reduced, particularly in men, and was independently associated with male sex, younger age, and higher myocardial native T1. Native T1 emerged as the principal imaging correlate of impaired performance, whereas conventional structural measures contributed little explanatory value. In subgroup analyses, higher resting lactate, but not peak exercise lactate, identified a metabolic phenotype associated with reduced aerobic capacity, supporting the concept that subtle metabolic and diffuse myocardial tissue alterations, rather than overt structural remodeling, may underlie persistent exercise limitation long after infection.

## Funding

German Ministry of Education and Research via the German Centre for Cardiovascular Research (DZHK) partner site RheinMain to EN, VP, SM. MH. Cardio-Pulmonary Institute (CPI), EXC 2026, Project ID: 390649896.

## Author contributions

**Monika Rozewicz-Juraszek:** Writing – review & editing, Writing – original draft, Investigation. **Stephan Mueller:** Writing – review & editing, Software, Resources, Funding acquisition, Formal analysis, Data curation. **Midjisuren Ganbat:** Writing – review & editing, Investigation. **Carlos Rodriguez Bolanos:** Writing – review & editing, Investigation. **Anna Klement:** Writing – review & editing, Visualization, Methodology, Investigation. **Sonia Antoñana Ugalde:** Writing – review & editing, Methodology, Investigation. **Martin Halle:** Writing – review & editing, Resources, Methodology, Investigation, Formal analysis. **Eike Nagel:** Writing – review & editing, Writing – original draft, Visualization, Validation, Supervision, Software, Resources, Project administration, Methodology, Investigation, Funding acquisition, Formal analysis, Data curation, Conceptualization. **Valentina O. Puntmann:** Writing – review & editing, Writing – original draft, Visualization, Validation, Supervision, Software, Resources, Project administration, Methodology, Investigation, Funding acquisition, Formal analysis, Data curation, Conceptualization.

## Ethics approval and consent

The study was approved by the Ethics Committee of the Goethe University Hospital Frankfurt, Germany (No: 463/16); Impression Study NCT04444128. All procedures were performed in accordance with the Declaration of Helsinki and the International Conference on Harmonization in Good Clinical Practice. Written informed consent was provided by each patient.

## Declaration of competing interests

German Ministry of Education and Research via the German Centre for Cardiovascular Research (DZHK) partner site RheinMain to EN, VP, SM, MH. Cardio-Pulmonary Institute (CPI), EXC 2026, Project ID: 390649896. The authors declare that they have no competing interests.
