# Supplementary material for: Native T1 is independently associated with aerobic exercise capacity in long-term follow-up after mild initial COVID-19 disease (Impression COVID&Heart Study)
Source: J Cardiovasc Magn Reson. 2026 Apr 15;28(1):102725. doi: 10.1016/j.jocmr.2026.102725 (PMC13246307; doi:10.1016/j.jocmr.2026.102725)
Supplement: Supplementary Tables S1 — Supplementary material [file mmc1.docx]

**Supplementary material**

**Full Title: Native T1 is independently associated with aerobic exercise capacity in long-term follow-up after mild initial COVID-19 disease (Impression COVID&Heart Study)**

**Short title: Native T1 and long-term aerobic exercise capacity after COVID-19**

**Authors: Monika Rozewicz-Juraszek, MD1, Stephan Mueller, PhD2, Midjisuren Ganbat, MD1, Carlos Rodriguez Bolanos, MD1, Anna Klement, MD; Sonia Antoñana Ugalde1, MD; Martin Halle MD2, Eike Nagel, MD PhD1,4, Valentina Puntmann, MD PhD1,4**

**Supplementary Statistical Methods**

Predicted VO₂peak values were calculated using reference equations derived from the SHIP cohort [1] and, in sensitivity analyses, from the FRIEND registry [2,3].

The following variables were specified a priori for inclusion in multivariable models based on prior literature and physiological relevance: age, sex, BMI, native T1, native T2, LV-EDVi, and left atrial area. These variables were selected to reflect established determinants of aerobic capacity, myocardial tissue characteristics, and cardiac preload phenotype. In sex-stratified analyses, sex was omitted from the model.

Univariable regression analyses using FRIEND-derived %-predicted VO₂peak are reported in Tables S1–S3, and corresponding multivariable models in Tables S4–S6. False discovery rate (Benjamini–Hochberg) correction was applied to univariable analyses to account for multiple testing, with q-values reported alongside p-values.

Model assumptions were assessed using standard diagnostic procedures, including inspection of residual distributions and assessment of collinearity. Scatterplots with ordinary least-squares regression lines and 95% confidence intervals were generated for visualisation of associations.

| *Variable* | *Rationale* |
| --- | --- |
| *Age* | *Previous knowledge (even though accounted for in the reference values; eliminated in sex-stratified analyses)* |
| *Sex* | *Previous knowledge (even though accounted for in the reference values; eliminated in sex-stratified analyses)* |
| *Body mass index (BMI)* | *Previous knowledge (even though accounted for in the reference values)* |
| *T1* | *Prespecified imaging biomarker of interest; native T1 reflects diffuse myocardial tissue alterations and was the primary CMR parameter under investigation.* |
| *T2* | *Prespecified imaging biomarker of interest; native T2 reflects myocardial oedema and was included as a complementary tissue characterisation parameter.* |
| *LV end-diastolic volume indexed (LV-EDVi)* | *Previous knowledge. To account for baseline LV pump phenotype (a major determinant of oxygen delivery), we prespecified LV-EDVi as a covariate, as it reflects cardiac size/preload reserve and is mechanistically linked to stroke volume and VO₂max.* |
| *Left atrial area (LAA)* | *Highly sensitive parameter for increased preload, ventricular stiffness, reduced relaxation* |

**VO₂max Prediction Equations**

**SHIP (Study of Health in Pomerania) Equation**[1]
Predicted VO₂max (mL·kg⁻¹·min⁻¹) was calculated using a regression model based on the SHIP reference cohort with age categories, sex, and BMI category (BMI ≤25 vs >25):

$$\begin{matrix} \text{VO}_{2\max} & =47.7565-0.9880\times\text{Age\_code}-0.2356\times\text{Age\_code}^{2}-8.8697\times\text{Sex\_code}+2.3597\times\text{BMI\_code} \\ & -2.0308\times(\text{Age\_code}\times\text{BMI\_code})-3.7405\times(\text{Sex\_code}\times\text{BMI\_code}) \\ & +0.2512\times(\text{Age\_code}\times\text{Sex\_code})+1.3797\times(\text{Age\_code}\times\text{Sex\_code}\times\text{BMI\_code}) \end{matrix}$$

Where **Sex_code = 1 for male, 2 for female**, **BMI_code = 0 for BMI ≤25, 1 for BMI >25**, and **Age_code** corresponds to predefined age bands.
This model reflects the influence of age, sex, BMI, and their interactions in the SHIP reference population.

**FRIEND (Fitness Registry and the Importance of Exercise National Database) Equation**[2,3]
Predicted maximal oxygen uptake (VO₂max, mL·kg⁻¹·min⁻¹) was calculated as:

$$\text{VO}_{2\max}=79.9-0.39\times\text{Age (years)}-13.7\times\text{Sex}-0.28\times\text{Weight (kg)}$$

Where **Sex = 0 for male, 1 for female**.
This equation was originally derived from a large registry of healthy adults and is widely used for normal reference standards.

**Supplementary Results - Tables**

**Table S1**. Univariate analysis versus %-predicted VO₂peak using the FRIEND dataset [2,3] for the full cohort (N=132).

| **Characteristic** | **Β** | **95% CI** | **p-value** | **q-value***^1^* |
| --- | --- | --- | --- | --- |
| Age (years) | 0.71 | 0.47, 0.95 | **<0.001** | <0.001 |
| Body mass index (kg/m²) | 0.01 | -0.72, 0.74 | >0.9 | >0.9 |
| Sex | 16 | 11, 22 | **<0.001** | <0.001 |
| LVEF (%) | 0.17 | -0.31, 0.65 | 0.5 | 0.6 |
| LV EDVi (mL/m²) | -0.20 | -0.43, 0.02 | 0.077 | 0.2 |
| LV SVi (mL/m²) | -0.28 | -0.67, 0.11 | 0.2 | 0.2 |
| LV mass index (g/m²) | -0.37 | -0.65, -0.09 | **0.010** | 0.029 |
| RVEF (%) | 0.38 | -0.13, 0.88 | 0.14 | 0.2 |
| Left atrial area (cm²) | -0.23 | -0.94, 0.48 | 0.5 | 0.6 |
| E/e′ (ratio) | 2.1 | 0.25, 3.9 | **0.026** | 0.062 |
| Native T1 (ms) | -0.13 | -0.23, -0.03 | **0.010** | 0.029 |
| Native T2 (ms) | -0.47 | -2.5, 1.5 | 0.6 | 0.7 |
| Abbreviation: CI = Confidence Interval | | | | |
| *^1^* False discovery rate correction for multiple testing | | | | |

**Table S2**. Univariate analysis versus %-predicted VO₂peak using the FRIEND dataset [2,3] for the male cohort (N=64).

| **Characteristic** | **Β** | **95% CI** | **p-value** | **q-value***^1^* |
| --- | --- | --- | --- | --- |
| Age (years) | 0.58 | 0.29, 0.87 | **<0.001** | 0.001 |
| Body mass index (kg/m²) | 0.32 | -0.63, 1.3 | 0.5 | 0.6 |
| LVEF (%) | 0.41 | -0.41, 1.2 | 0.3 | 0.5 |
| LV EDVi (mL/m²) | 0.12 | -0.19, 0.43 | 0.5 | 0.6 |
| LV SVi (mL/m²) | 0.26 | -0.23, 0.75 | 0.3 | 0.5 |
| LV mass index (g/m²) | 0.23 | -0.17, 0.62 | 0.3 | 0.5 |
| RVEF (%) | -0.09 | -0.83, 0.64 | 0.8 | 0.8 |
| Left atrial area (cm²) | 0.74 | -0.17, 1.6 | 0.11 | 0.4 |
| E/e′ (ratio) | 1.5 | -0.70, 3.7 | 0.2 | 0.5 |
| Native T1 (ms) | -0.16 | -0.27, -0.04 | **0.007** | 0.037 |
| Native T2 (ms) | -0.99 | -3.7, 1.7 | 0.5 | 0.6 |
| Abbreviation: CI = Confidence Interval | | | | |
| *^1^* False discovery rate correction for multiple testing | | | | |

**Table S3**. Univariate analysis versus %-predicted VO₂peak using the FRIEND dataset [2,3] for the female cohort (N=68).

| **Characteristic** | **Β** | **95% CI** | **p-value** | **q-value***^1^* |
| --- | --- | --- | --- | --- |
| Age (years) | 0.80 | 0.48, 1.1 | **<0.001** | <0.001 |
| Body mass index (kg/m²) | 0.83 | -0.15, 1.8 | 0.10 | 0.3 |
| LVEF (%) | -0.10 | -0.62, 0.43 | 0.7 | 0.9 |
| LV EDVi (mL/m²) | 0.03 | -0.31, 0.37 | 0.9 | 0.9 |
| LV SVi (mL/m²) | -0.23 | -0.80, 0.34 | 0.4 | 0.6 |
| LV mass index (g/m²) | 0.05 | -0.50, 0.61 | 0.9 | 0.9 |
| RVEF (%) | -0.52 | -1.2, 0.21 | 0.2 | 0.3 |
| Left atrial area (cm²) | 0.55 | -0.52, 1.6 | 0.3 | 0.5 |
| E/e′ (ratio) | 2.0 | -0.47, 4.5 | 0.11 | 0.3 |
| Native T1 (ms) | -0.15 | -0.30, -0.01 | **0.036** | 0.2 |
| Native T2 (ms) | -1.3 | -3.9, 1.2 | 0.3 | 0.5 |
| Abbreviation: CI = Confidence Interval | | | | |
| *^1^* False discovery rate correction for multiple testing | | | | |

**Table S4**: Multivariate analysis versus %-predicted VO₂peak using the FRIEND dataset [2,3] for the full cohort (N=132).

| **Characteristic** | **Β** | **95% CI** | **p-value** | **q-value***^1^* |
| --- | --- | --- | --- | --- |
| Age (years) | 0.72 | 0.52, 0.92 | **<0.001** | <0.001 |
| Sex | 24 | 18, 29 | **<0.001** | <0.001 |
| Body mass index (kg/m²) | 1.1 | 0.51, 1.8 | **<0.001** | <0.001 |
| LV EDVi (mL/m²) | 0.27 | 0.06, 0.48 | **0.014** | 0.020 |
| Left atrial area (cm²) | 0.45 | -0.17, 1.1 | 0.2 | 0.2 |
| Native T1 (ms) | -0.21 | -0.30, -0.12 | **<0.001** | <0.001 |
| Native T2 (ms) | -0.71 | -2.3, 0.90 | 0.4 | 0.4 |
| R² | 0.51 |  |  |  |
| Adjusted R² | 0.48 |  |  |  |
| AIC | 1,249 |  |  |  |
| BIC | 1,276 |  |  |  |
| Abbreviation: CI = Confidence Interval | | | | |
| *^1^* False discovery rate correction for multiple testing | | | | |

**Table S5**. Multivariate analysis versus %-predicted VO₂peak using the FRIEND dataset [2,3] for the male cohort (N=64).

| **Characteristic** | **Β** | **95% CI** | **p-value** | **q-value***^1^* |
| --- | --- | --- | --- | --- |
| Age (years) | 0.65 | 0.36, 0.94 | **<0.001** | <0.001 |
| Body mass index (kg/m²) | 0.81 | -0.11, 1.7 | 0.083 | 0.12 |
| LV EDVi (mL/m²) | 0.29 | 0.00, 0.59 | 0.052 | 0.10 |
| Left atrial area (cm²) | 0.56 | -0.34, 1.5 | 0.2 | 0.3 |
| Native T1 (ms) | -0.23 | -0.35, -0.11 | **<0.001** | <0.001 |
| Native T2 (ms) | 0.57 | -1.9, 3.1 | 0.6 | 0.6 |
| R² | 0.38 |  |  |  |
| Adjusted R² | 0.33 |  |  |  |
| AIC | 620 |  |  |  |
| BIC | 638 |  |  |  |
| Abbreviation: CI = Confidence Interval | | | | |
| *^1^* False discovery rate correction for multiple testing | | | | |

**Table S6**. Multivariate analysis versus %-predicted VO₂peak using the FRIEND dataset [2,3] for the female cohort (N=68).

| **Characteristic** | **Β** | **95% CI** | **p-value** | **q-value***^1^* |
| --- | --- | --- | --- | --- |
| Age (years) | 0.85 | 0.55, 1.2 | **<0.001** | <0.001 |
| Body mass index (kg/m²) | 1.5 | 0.61, 2.4 | **0.001** | 0.004 |
| LV EDVi (mL/m²) | 0.25 | -0.07, 0.57 | 0.12 | 0.14 |
| Left atrial area (cm²) | 0.68 | -0.24, 1.6 | 0.15 | 0.15 |
| Native T1 (ms) | -0.19 | -0.32, -0.05 | **0.008** | 0.015 |
| Native T2 (ms) | -2.1 | -4.3, 0.18 | 0.071 | 0.11 |
| R² | 0.44 |  |  |  |
| Adjusted R² | 0.39 |  |  |  |
| AIC | 636 |  |  |  |
| BIC | 655 |  |  |  |
| Abbreviation: CI = Confidence Interval | | | | |
| *^1^* False discovery rate correction for multiple testing | | | | |

**References**

1. Koch B, Schäper C, Ittermann T, Spielhagen T, Dörr M, Völzke H, et al. Reference values for cardiopulmonary exercise testing in healthy volunteers: the SHIP study. Eur Respir J. 2008;33:389–97.

2. Myers J, Kaminsky LA, Lima R, Christle JW, Ashley E, Arena R. A Reference Equation for Normal Standards for VO2 Max: Analysis from the Fitness Registry and the Importance of Exercise National Database (FRIEND Registry). Prog Cardiovasc Dis. 2017;60:21–9.

3. Kaminsky LA, Arena R, Myers J, Peterman JE, Bonikowske AR, Harber MP, et al. Updated Reference Standards for Cardiorespiratory Fitness Measured with Cardiopulmonary Exercise Testing. Mayo Clin Proc. 2022;97:285–93.
